# Predictive variables and diagnostic performance of cross-sectional models for hypertension detection: a systematic review

**DOI:** 10.3389/fcvm.2025.1713531

**Published:** 2025-11-28

**Authors:** Víctor Juan Vera-Ponce, Fiorella E. Zuzunaga-Montoya, Jhosmer Ballena-Caicedo, Carmen Inés Gutierrez De Carrillo, Darwin A. León-Figueroa, Mario J. Valladares-Garrido

**Affiliations:** 1 Facultad de Medicina (FAMED), Universidad Nacional Toribio Rodríguez de Mendoza de Amazonas (UNTRM), Amazonas, Perú; 2 Universidad Continental, Lima, Perú; 3 Facultad de Medicina Humana, Universidad de San Martín de Porres, Chiclayo, Peru; 4 EpiHealth Research Center for Epidemiology and Public Health, Lima, Peru; 5 Escuela de Medicina Humana, Universidad Señor de Sipán, Chiclayo, Peru

**Keywords:** hypertension, risk assessment, cross-sectional studies, predictive value of tests, machine learning, risk factors, diagnosis

## Abstract

**Introduction:**

Hypertension (HTN) affects approximately 1.3 billion people worldwide, nearly half of whom remain undiagnosed, underscoring the urgent need for efficient diagnostic models to enable timely detection. This study aimed to identify and critically evaluate cross-sectional predictive models developed for diagnosing hypertension in adults globally.

**Methods:**

A systematic review was conducted in accordance with the PRISMA (Preferred Reporting Items for Systematic Reviews and Meta-Analyses) and TRIPOD guidelines, searching MEDLINE/PubMed, Scopus, Web of Science, and EMBASE from January 2000 to March 2024. We included observational studies that developed or validated cross-sectional models for adult HTN detection. The risk of bias was assessed using the PROBAST (Prediction Model Risk of Bias Assessment Tool).

**Results:**

Eight studies met the inclusion criteria, predominantly from Asia (four from China, one from Korea, one from Qatar, one from the United Arab Emirates, and one from Bangladesh). The models demonstrated acceptable discriminatory capacity [area under the receiver operating characteristic curve (AUC) 0.70-0.89], with minimal differences between traditional statistical approaches (logistic regression) and machine learning methods. The most consistent predictors were age (present in >90% of models), body mass index (85%-90%), sex/gender (75%-80%), and diabetes (70%-75%). Laboratory biomarkers provided only marginal improvements in predictive performance (AUC increase of +0.02-0.04) compared with models based exclusively on clinical variables, raising concerns about their cost-effectiveness. Notably, no predictive models were identified for Latin American populations, despite the region's high prevalence of HTN. This absence highlights a critical research gap.

**Conclusion:**

Cross-sectional predictive models represent valuable tools for the detection of HTN, with simplified clinical models performing nearly as well as more complex approaches. Future research should prioritize the development of models tailored to underrepresented populations, integrating social determinants of health and adopting accessible formats to facilitate implementation in resource-limited settings.

## 1 Introduction

Hypertension (HTN) constitutes one of the main contemporary health challenges, affecting more than 1.3 billion people globally, approximately 34% of the world's adult population (1, 2). This silent condition represents the principal modifiable risk factor for cardiovascular disease and contributes substantially to the global mortality burden, being responsible for approximately 8.5 million annual deaths (3). Its prevalence continues to increase, particularly in low and middle-income countries, where nearly 75% of affected individuals reside, imposing a disproportionate burden on already overwhelmed health systems (1, 4).

Despite its epidemiological significance, approximately 46% of adults with HTN are unaware of their condition, which underscores the urgent need to improve early detection strategies (5). The World Health Organization (WHO) estimates that only 42% of individuals with HTN receive treatment, and merely 21% achieve adequate blood pressure control (2). The high associated economic costs compound this concerning epidemiological scenario, estimated at more than $370 billion annually worldwide, which constitute 10%–15% of total healthcare expenditure in many countries (6).

While prognostic models predict future HTN development (5, 7), cross-sectional models address a more immediate need: detecting existing undiagnosed cases. These models identify individuals with a high probability of current HTN through readily available clinical and demographic data, without requiring direct blood pressure measurement. This approach offers practical advantages for population screening, as it reduces the costs associated with universal blood pressure monitoring, enables risk stratification to prioritize diagnostic resources, and extends the reach of screening to populations with limited access to healthcare. Multiple cross-sectional models have been developed worldwide; yet, considerable methodological heterogeneity exists, making it difficult to determine which models demonstrate adequate validity and which predictors are most reliable. Critical synthesis of these models is needed to inform evidence-based screening strategies and reduce the substantial burden of undiagnosed HTN, which affects nearly half of all hypertensive individuals globally.

Therefore, the present systematic review (SR) aims to identify and critically evaluate cross-sectional predictive models for treating HTN in adults worldwide. As secondary objectives, the most frequently employed predictor variables in these models will be explored, and their methodological quality will be evaluated using standardized tools. This synthesis will enable the identification of valid diagnostic tools for diverse contexts, indicating those that require more evidence before their widespread implementation, thereby reducing existing knowledge gaps and guiding the development of more effective predictive instruments that can be generalized to diverse populations.

## 2 Methodology

### 2.1 Study design

A SR focused on studies that developed or validated cross-sectional predictive models for treating HTN in the adult population. This review followed the guidelines of PRISMA 2020 (Preferred Reporting Items for Systematic Reviews and Meta-Analyses) (8), as well as the methodological recommendations of PRISMA-DTA (PRISMA for diagnostic test accuracy studies) and the Cochrane Prognosis Methods Group for SRs of prediction models (9) (Supplementary Material 1). Additionally, the guidance from the TRIPOD guidelines for evaluating predictive model studies was considered (10). This review did not include a meta-analysis due to the methodological and population heterogeneity of the studies; however, a structured and tabulated narrative synthesis was performed.

### 2.2 Search strategy

The bibliographic search was conducted in the following databases: MEDLINE/PubMed, Scopus, Web of Science, and EMBASE, selected for their multidisciplinary coverage, international scope, and their utility for SRs according to Cochrane Library recommendations (11). The SciELO database was not directly used since its contents are mostly included in Web of Science. MeSH terms and keywords were employed and combined using Boolean operators. The terms used included: (“Hypertension” OR “High Blood Pressure”) AND (“Prediction Model” OR “Risk Score” OR “Nomogram” OR “Machine Learning” OR “Diagnostic Prediction”) AND (“Cross-sectional” OR “Screening”) AND (“Adult”). The search was restricted to studies published between January 1, 2000, and March 15, 2024, without language limitation. The complete strategy is included as Supplementary Material 2.

### 2.3 Selection criteria

Observational studies (mainly cross-sectional or cohort studies analyzed cross-sectionally) that had developed or validated diagnostic or predictive cross-sectional models for HTN detection in adult populations (≥18 years), regardless of sex, clinical context, or country, were included. The models had to include clinical, sociodemographic, anthropometric, or lifestyle predictor variables, and report at least one performance metric [area under the receiver operating characteristic curve (AUC), sensitivity, specificity, PV, etc.]. Studies employing any valid operational definition of HTN according to national or international guidelines (≥140/90 mmHg or ≥130/80 mmHg, with or without medication use) were accepted (12, 13).

Articles that did not present a prediction model (such as narrative reviews, editorials, letters, clinical cases), exclusively prognostic studies (future prediction), studies that used direct blood pressure measurement as the main predictor (as they are not applicable as screening tools), as well as those conducted exclusively in pediatric populations, pregnant women, or hospitalized patients with severe acute pathologies were excluded. Articles that did not report validation metrics or did not present results applicable to cross-sectional diagnosis were also excluded.

### 2.4 Study selection process

Search results were imported into Rayyan QCRI software, which was used for reference management and the automatic removal of duplicates. Subsequently, two reviewers independently and blindly evaluated all titles and abstracts to determine their eligibility. Studies that met the criteria were retrieved in full text for detailed evaluation. Discrepancies between reviewers were resolved through discussion and consensus. In cases of persistent disagreement, the opinion of a third reviewer was sought to make the final decision.

### 2.5 Data extraction

Two reviewers independently and in parallel performed data extraction using a standardized template in Microsoft Excel 2023. From each study, the following was extracted: author and year of publication, country or region, study design, target population, sample size, database or data source, predictor variables used, HTN definition, statistical method or type of algorithm applied (regression, machine learning, etc.), internal or external validation, and model performance metrics (AUC, sensitivity, specificity, predictive values). Additionally, it was recorded whether an implementable model (score, nomogram, app, etc.) was presented. The information was cross-verified by both reviewers, and any discrepancy was resolved by consensus.

### 2.6 Risk of bias assessment

The risk of bias of included studies was assessed using the PROBAST tool (Prediction model Risk Of Bias Assessment Tool) (14), recommended by the Cochrane group for diagnostic prediction model studies. This tool evaluates four domains: participants, predictors, outcome, and statistical analysis, classifying the risk of bias as “high”, “low”, or “uncertain”. Two independent reviewers performed this assessment, and any discrepancy was resolved by consensus or a third evaluator. The results of the risk of bias assessment were considered in the final interpretation of the findings and the narrative synthesis, and are presented in tabulated form.

### 2.7 Qualitative analysis

Given that the included studies presented marked heterogeneity in their methodological designs, target populations, predictor variables, and reported statistical metrics, a comparative qualitative analysis was chosen instead of a meta-analysis. This decision was based on the diversity of analytical approaches (logistic regression, machine learning, nomograms, population scores), definitions of HTN (≥140/90 mmHg vs. ≥130/80 mmHg), and contextual characteristics such as geographic region and population inclusion criteria, which made a uniform quantitative synthesis of predictive performances impossible.

The qualitative analysis involved extracting and systematizing key information through structured comparative tables. The first table summarizes the general methodological aspects of each study, including country of origin, type of design (derivation, validation, population-based), target population, sample size, data source, and operational definition of HTN used. A second table details the coding and type of each predictor variable included in the models, allowing the identification of common patterns and relevant differences regarding predictor selection and operationalization.

Subsequently, the discriminative performance of the models was compiled in terms of C-statistic or AUC, sensitivity, and specificity, along with the type of predictive tool employed (manual score, probability index, nomogram, machine learning models).

## 3 Results

### 3.1 Article selection flow

**Figure 1:**
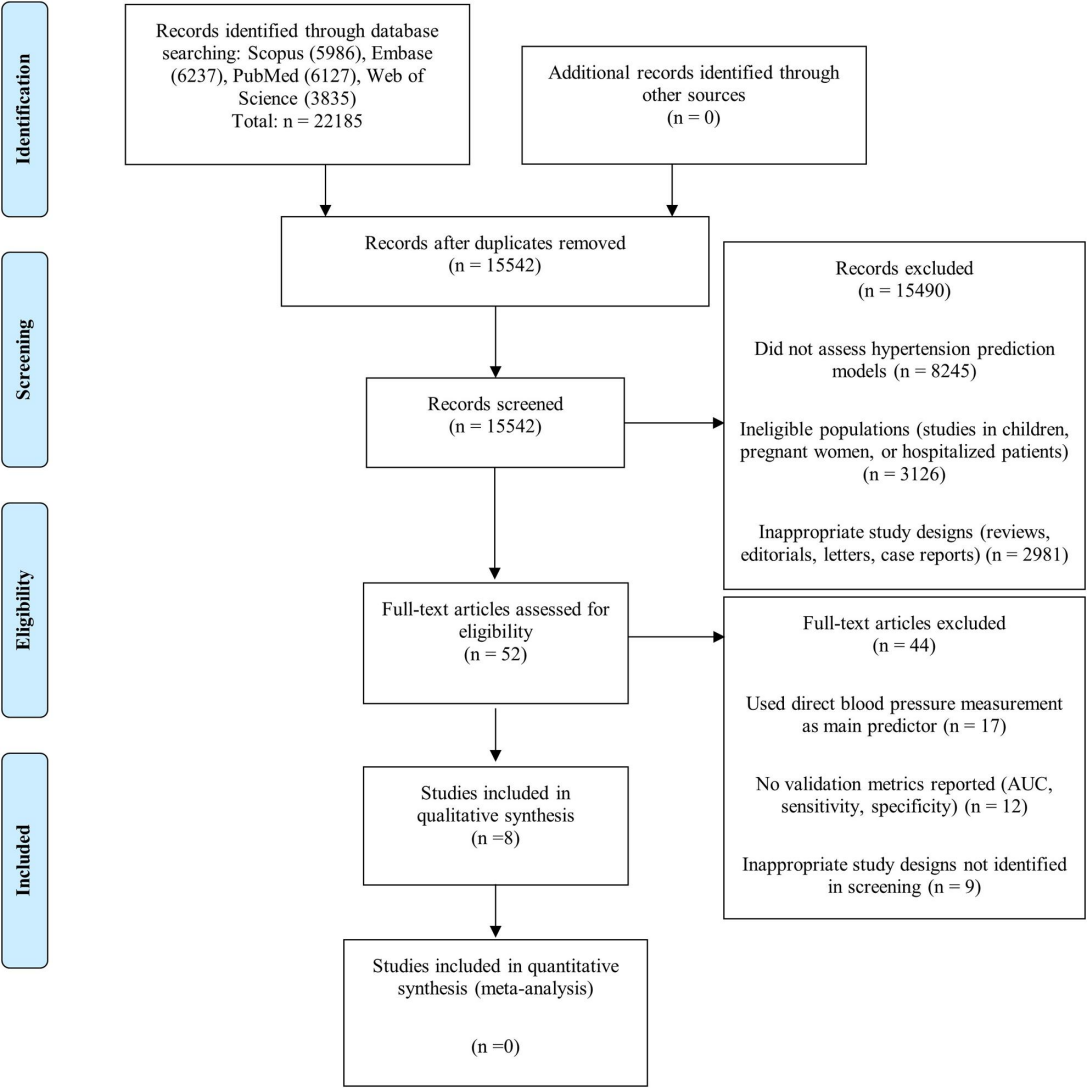
Flowchart of study selection.

From 22,185 initial records identified in four databases (Scopus, Embase, PubMed, and Web of Science), 6,643 duplicates were eliminated, resulting in 15,542 records for review. After initial screening, 15,490 records were excluded mainly because they did not evaluate HTN predictive models (8,245), studied ineligible populations (3,126), presented inappropriate designs (2,981), were prognostic rather than diagnostic models (914), or contained incomplete data (224). Of the 52 articles evaluated in full text, 44 were excluded for using direct blood pressure measurement as the main predictor (17), not reporting validation metrics (12), having inappropriate designs (9), or presenting duplicate analyses (6). Finally, eight studies met all inclusion criteria for qualitative synthesis (15–22). The complete flowchart can be seen in Figure 1.

### 3.2 Characteristics of included studies

This SR included eight studies published between 2014 and 2024, which developed and/or validated cross-sectional predictive models for the diagnosis of HTN in the adult population (Table 1). Geographically, the studies were conducted mainly in Asia, covering countries such as China (4 studies) (16, 19–21), South Korea (15), Qatar (17), the United Arab Emirates (18), and Bangladesh (22).

**Table 1:** Summary of the methodological characteristics of included studies on HTN predictive models.

First author and year	Country/Region	Study design	Target population	Sample size	Data source	HTN definition
Choo (2014)	South Korea	Cross-sectional with external validation	Adults ≥20 years without previous diagnosis	4,382 (validation)	KNHANES Survey	≥140/90 mmHg without previous diagnosis
Ren (2020)	China	Cross-sectional with internal validation	Adults in physical check-up center	68,980	University hospital	≥130/80 mmHg (AHA 2017)
AlKaabi (2020)	Qatar	Cross-sectional with cross-validation (5-fold)	Adults ≥18 years (Qataris and residents)	987	Qatar Biobank	≥140/90 mmHg or medication
Mahmoud (2021)	UAE	Population-based cross-sectional with internal validation	Adults ≥18 years	2,533	Dubai Household Survey	≥140/90 mmHg or self-report with medication
Jiang (2022)	China	Cross-sectional with internal validation	Adults ≥15 years	3,147 (1,573 derivation/1,574 validation)	Shanghai Survey	≥140/90 mmHg or medication
Ji (2022)	China	Cross-sectional with internal validation	Multiethnic adults ≥18 years	4,287,407	National physical examination	≥140/90 mmHg or medication
Yu (2023)	China	Cross-sectional with internal and external validation	Han and Yugur (20–80 years)	9,699	China National Health Survey	≥140/90 mmHg or medication
Islam (2024)	Bangladesh	Cross-sectional with internal validation	Adults ≥35 years	6,965	BDHS 2017–18	≥140/90 mmHg or medication

In methodological terms, all studies used a cross-sectional design and applied some type of predictive model validation. Six studies performed only internal validation through data partitioning or cross-validation strategies: Ren (16), AlKaabi (17), Mahmoud (18), Jiang (20), Ji (19), and Islam (22). Choo's study (15) performed only external validation in an independent Korean population. Yu's study (21) was the only one to perform both internal and external validation, demonstrating model performance across training, internal validation, and external validation datasets. The sample size varied significantly, from 987 participants (AlKaabi) (17) to more than 4.2 million (Ji) (19).

Regarding the operational definition of HTN, seven studies used the classic diagnostic threshold of ≥140/90 mmHg or self-reported use of antihypertensive medications. Only Ren et al.'s study (16) applied the criterion of ≥130/80 mmHg. Additionally, there was variability in data collection methods, from population surveys and hospital databases to national administrative records.

Five studies developed applicable tools, including nomograms, self-administered scores, and interactive web tools, for practical implementation. This represents an important advance toward the clinical or community use of the models, particularly in populations without immediate access to blood pressure measurement. It should be noted that machine learning-based models, although promising, generally do not present versions usable by the public (for example, in the form of an app or formula), which may limit their direct implementation in low-resource contexts.

### 3.3 Analysis of HTN predictive models

Various studies have developed predictive models to estimate HTN risk, implementing different formats and methodologies (Table 2). Choo (15) presented a self-administered score through logistic regression with KNHANES data, achieving an AUC of 0.757 with 75% sensitivity and 65% specificity in external validation. This model stands out for its simplicity and direct applicability by users without the need for clinical tests. In contrast, Ren (16) developed a probability index based on logistic regression and validated with Random Forest, achieving an AUC of 0.758 in the validation set without reporting sensitivity or specificity, requiring greater computational processing for its implementation.

**Table 2:** Predictive models for HTN: statistical methods and performance metrics.

First author and year	Statistical method	Tool type	AUC/C-statistic	Sensitivity	Specificity	Additional comment
Choo (2014)	Logistic regression	Self-administered score	0.757 (external validation)	75%	65%	External validation with KNHANES survey. Max score = 11
Ren (2020)	Logistic regression	Probability index (POD)	0.758 (test set)	Not reported	Not reported	Model validated with logistic regression and random forest
AlKaabi (2020)	Logistic regression, Random Forest, Decision tree	Risk score (ML)	0.85 (logistic), 0.869 (RF), 0.799 (tree)	81.1%–82.1%	Not reported	Machine learning was used on Qatar Biobank data
Mahmoud (2021)	Logistic regression (score)	Population score (manual, simple)	0.77 (total), 0.83 (Emirati), 0.74 (non-Emirati)	81.0% (total)	56.0% (total)	Model internally validated with optimal cutoff ≥25
Jiang (2022)	Logistic regression (score)	Screening tool by score	0.817	83.40%	64.30%	Simple variables, no laboratory tests needed
Ji (2022)	XGBoost (ML) and comparative with LR	ML models (XGBoost, RF, ANN, etc.)	0.893 (no lab), 0.894 (semi-lab)	61.8% (no lab)	91.3% (no lab)	XGBoost was the best algorithm with large sample size
Yu (2023)	LASSO + logistic regression	Nomogram + website	0.802 (training), 0.789 (internal validation), 0.829 (external)	Not reported	Not reported	Score with risk classification (low, intermediate, high). Web visualization
Islam (2024)	LASSO + logistic regression	Nomogram	0.729 (training), 0.715 (validation)	89.4% (training), 87.8% (validation)	Not reported	First nomogram model for Bangladesh

The application of advanced machine learning algorithms is evidenced in AlKaabi (17), who compared different methods to develop an automated risk score in Qatar. Their Random Forest model achieved excellent performance (AUC = 0.869, sensitivity 82.1%, PPV 81.4%), outperforming logistic regression (AUC = 0.85). In the same line of methodological innovation, Ji (19) evaluated multiple algorithms (XGBoost, Random Forest, neural networks) in a study with more than 4 million participants in China, where XGBoost obtained the best performance with an AUC of 0.893 in the model without laboratory values and 0.894 with biochemical variables, achieving high specificity (91.3%) at the cost of lower sensitivity (61.8%).

Some researchers have prioritized accessibility and clinical applicability. Mahmoud (18) developed a manual population score from the Dubai Household Survey, with an overall AUC of 0.77 (0.83 in Emiratis, 0.74 in non-Emiratis), 81% sensitivity and 56% specificity, specifically designed for community prevention programs. Similarly, Jiang (19) constructed a score-based screening tool through logistic regression that does not require laboratory tests, achieving an AUC of 0.817, 83.4% sensitivity, and 64.3% specificity, ideal for implementation in primary care.

Nomograms represent an innovative visual format, as demonstrated by Yu's work (21), who developed a model accompanied by an interactive web platform for Han and Yugur populations in China. Based on logistic regression with LASSO selection, this model showed excellent discrimination (AUC of 0.802 in training, 0.789 in internal validation, and 0.829 in external validation), offering a risk-level classification supported by clinical decision analysis. Islam et al. (22) followed a similar approach for Bangladesh, using data from BDHS 2017–18 and LASSO-logistic regression methodology, achieving an AUC of 0.729 in training and 0.715 in validation, with high sensitivity (89.4% and 87.8%, respectively) and good calibration demonstrated through decision and clinical impact curves.

These studies reveal an evolution from simple, self-calculable models to more sophisticated artificial intelligence systems and digital platforms. Predictive performance has progressively improved, reaching AUC values above 0.85 in the most recent machine learning-based models. At the same time, classic approaches of logistic regression and nomograms maintain acceptable performance (AUC 0.7–0.8) with greater interpretability and practical applicability. The choice of tool type will depend on the application context, available resources, and specific population characteristics, all of which are valuable contributions to early detection and prevention of HTN.

### 3.4 Predictor variables used and their measurement form

The reviewed HTN predictive models presented important variations in the variables included, both in number and how they were coded (Table 3). In most studies, age was included as a key predictor, either as a categorical variable (15, 20, 22) or continuous (16, 19, 21), reflecting the well-documented relationship between aging and increased HTN risk. Similarly, sex was used as a dichotomous variable in almost all models, considering male sex as the higher risk category.

**Table 3:** Predictor variables used in HTN models.

Study	Age	Sex	Body mass index	Abdominal waist	Physical activity	Smoking	Alcohol	Diabetes	Lipids	HTN family history	Education	Fruits/Vegetables	Others
Choo (2014)	Categorical (<35, 35–44, 45–54, ≥55)	Dichotomous (F/M)	–	Categorical (<84/77, 84–89.9/77–84.9, ≥90/85)	–	–	drinks/week <7, 7–34.9, ≥35	–	–	Dichotomous (Yes/No)	–	–	–
Ren (2020)	Continuous	Dichotomous (F/M)	Not directly. Weight and height used separately	–	–	–	–	–	Continuous (LDL, HDL, TG)	–	–	–	Urea, Creatinine
AlKaabi (2020)	Categorical (<50, ≥50)	Dichotomous (F/M)	–	Dichotomous (≥102/94 cm)	Dichotomous (≥150 min/week vs. <150)	Dichotomous (Yes/No)	–	Dichotomous (History of diabetes)	Dichotomous (History of high cholesterol)	Dichotomous (Yes/No) (specifically mother with HTN)	Categorical (low vs. secondary+)	Dichotomous (≥4 servings/day)	–
Mahmoud (2021)	Categorical (18–39, ≥40)	Dichotomous (F/M)	Categorical (normal, overweight, obesity)	–	–	–	–	Dichotomous	Dichotomous (dyslipidemia: Yes/No)	–	Categorical (low, secondary, tertiary)	–	–
Jiang (2022)	Categorical (<60, ≥60)	Dichotomous (F/M)	Categorical (<24, ≥24 kg/m^2^)	Dichotomous (≥90/85 cm by sex)	–	–	–	Dichotomous	Dichotomous (dyslipidemia: Yes/No)	Dichotomous (Yes/No)	Dichotomous (junior high school or below vs. high school or above)	Dichotomous (≥1 time/day)	–
Ji (2022)	Continuous	Dichotomous (F/M)	Continuous	Continuous	Categorical (exercise frequency)	Categorical (never smoked, quit smoking, smoking)	Frequency of consumption	Dichotomous	Continuous (total cholesterol, LDL, HDL, triglycerides)	Dichotomous (Yes/No)	–	–	SBP, DBP, Ethnicity, albumin, total bilirubin, EKG
Yu (2023)	Categorical (20–29, 30–39, 40–49, 50–59, 60–68, 70–80)	Dichotomous (F/M)	Categorical (<24, 24–27.9, ≥28 kg/m^2^)	–	Low, Moderate, High	Dichotomous (No/Ex or current)	Dichotomous (No, Ex/Current)	–	–	Categorical (FH0, FH1, FH2+)	Low, Medium, High	–	Ethnicity, annual income, current residence (urban/rural), marital status (single, married, divorced/widowed), FHH, Heart rate
Islam (2024)	Categorical (<65, ≥65)	Dichotomous (F/M)	Categorical (normal, underweight, overweight/obese)	–	Dichotomous (active/inactive)	Dichotomous (Yes/No)	–	Dichotomous	–	–	Categorical (No-education, Primary, Secondary, Higher)	–	Socioeconomic status, Region, Employment status (Yes/No), current residence (urban/rural), marital status (Never married, Married, Widowed, Divorced)

Body mass index (BMI) or related anthropometric measures were present in most models, though operationalized differently across studies. Only Ji (19) coded BMI as a continuous variable. Four studies treated BMI categorically: Mahmoud (18), Jiang (20), Yu (21), and Islam (22), differentiating between normal weight, overweight, and obesity using various cutoff points. Other studies used alternative anthropometric indicators: Ren (16) included height and weight as separate continuous variables rather than calculating BMI; AlKaabi (17) substituted BMI with a dichotomous abdominal obesity variable using sex-specific waist circumference cutoff points (≥102 cm in men or ≥94 cm in women); and Choo (15) used only waist circumference. This variability highlights different approaches to capturing body composition and central fat distribution as cardiovascular risk factors.

Physical activity was included dichotomously (active/inactive) or categorically (weekly frequency) in studies such as AlKaabi (17), Ji (19), and Islam (22). However, it was excluded in some final models because it did not reach significance (Mahmoud) (18). Regarding smoking, most studies used a dichotomous variable (current smoker: yes/no), although Ji (19) coded it in more detail using three categories (never smoked, quit smoking, smoking), allowing a more granular analysis of cumulative exposure effects.

Comorbidities such as diabetes mellitus and dyslipidemia were also frequent. Five studies included diabetes mellitus as a dichotomous variable: Mahmoud (18), AlKaabi (17), Ji (19), Jiang (20), and Islam (22). Dyslipidemia as a dichotomous variable was included in three studies: AlKaabi (17), Mahmoud (18), and Jiang (20). Ji's study (19) instead included lipid variables (total cholesterol, LDL, HDL, triglycerides) as continuous values, reflecting the richness of available clinical data. Similarly, Ren (16) included lipid markers as continuous variables without requiring prior categorization.

Family history of hypertension was a variable considered in five studies, with important differences in coding. Four studies employed a dichotomous version (yes/no): Choo (15), AlKaabi (17), Jiang (20), and Ji (19). Yu (21) stands out for having included a more detailed categorical approach by classifying it according to affected generations (FH0, FH1, FH2+), which provides additional prognostic value. The remaining three studies (Ren, Mahmoud, Islam) did not include family history, possibly due to lack of available data.

Education as a social determinant was included in five studies, with different coding approaches. Four studies coded education as an ordinal categorical variable (low, medium, high): Mahmoud (18), AlKaabi (17), Yu (21), and Islam (22). Jiang (20) used a dichotomous classification (junior high school or below vs. high school or above). These approaches allow capturing social gradients associated with cardiovascular risk (Table 3).

This heterogeneity in variable selection and coding reflects differences in data availability and the analytical priorities of each study. However, the most consistent predictors across models were age, sex, BMI (or waist circumference), diabetes, and dyslipidemia. The inclusion of social and behavioral factors such as physical activity, education, or diet is still variable, despite their recognized importance. These observations underscore the need to move toward more standardized, comparable, and applicable models for different population contexts, incorporating both clinical and social determinants of HTN (Table 3).

### 3.5 Explanation of risk of bias analysis (PROBAST)

The PROBAST tool assesses the methodological quality of prediction model studies in four main domains: participant selection, predictor measurement, outcome determination, and statistical analysis (Table 4). In our SR, most studies showed low risk of bias regarding participant selection and predictors, as they adequately defined their inclusion criteria and used accessible and clinically relevant variables. However, some studies such as Choo (15), Ren (16), and AlKaabi (17) presented elevated risk in the analysis domain, mainly due to the absence of external validation and the limited use of adjustment techniques to avoid overfitting or statistical biases.

**Table 4:** Risk of bias and applicability assessment of cross-sectional predictive models using the PROBAST tool.

Study	Objective	Participants	Predictors	Outcome	Analysis	Applicability participants	Predictors	Outcome	Risk of bias	Applicability
Choo (2014)	Validation	+	+	+	−	+	+	+	−	+
Ren (2020)	Derivation	+	+	+	−	+	+	+	−	+
AlKaabi (2020)	Derivation	+	+	+	−	+	+	+	−	+
Mahmoud (2021)	Derivation	+	+	+	+	+	+	+	+	+
Jiang (2022)	Derivation	+	+	+	+	+	+	+	+	+
Ji (2022)	Derivation	+	+	+	+	+	+	+	+	+
Yu (2023)	Derivation	+	+	+	+	+	+	+	+	+
Islam (2024)	Derivation	+	+	+	+	+	+	+	+	+

In contrast, more recent studies (Jiang, Ji, Yu, and Islam) (19–22) met all key methodological criteria, using modern techniques such as penalized regression (LASSO), internal or external validation, and calibration and clinical decision curves. All studies showed good applicability, meaning their models are potentially useful for clinical application in the target populations. This evaluation suggests that newer models are methodologically more robust, while older ones should be more cautiously interpreted due to analytical limitations.

## 4 Discussion

### 4.1 Main findings

This SR identified eight studies that developed cross-sectional predictive models for HTN detection, primarily in Asian populations (four from China, one from Korea, one from Qatar, one from the United Arab Emirates, and one from Bangladesh) covering countries such, with a notable absence of Latin American models. The models showed acceptable to good discriminative capacity (AUC 0.7–0.89), without substantial differences between traditional approaches (logistic regression) and machine learning. Consistent predictors were identified in most models: age (>90%), BMI (85%–90%), sex/gender (75%–80%), and diabetes (70%–75%). Laboratory biomarkers provided marginal performance improvements (AUC +0.02–0.04) compared to models based exclusively on accessible clinical variables, questioning their cost-effectiveness. Models with better practical implementation (nomograms, simplified scores) proved almost as effective as complex algorithms, favoring their applicability in resource-limited settings.

### 4.2 Interpretation of results in the context of existing literature

It is necessary to highlight that this SR specifically focuses on cross-sectional predictive models for the diagnosis of HTN, a methodological approach distinct from the traditional prognostic models based on longitudinal cohorts that predominate in the scientific literature. While the latter seeks to identify normotensive individuals with a higher risk of developing HTN in the future, our approach addresses an equally critical but different health need: the detection of people with already established but undiagnosed HTN. Various epidemiological surveys worldwide indicate that up to 30%–45% of hypertensive individuals are unaware of their condition (1, 2). This underscores the clinical relevance of cross-sectional diagnostic tools, conceptually similar to the widely used ADA Risk Test for diabetes (23). This fundamental methodological distinction should be considered when interpreting our findings in existing literature. Subsequently, comparisons will be established primarily with studies that evaluated prognostic models, analyzing parallels and divergences in predictor variables and analytical approaches.

The evolution of predictive models for HTN reflects the progressive methodological refinement observed in predictive medicine over the last decades. The most recent SRs, such as Schjerven et al. (7), have analyzed 53 studies, finding that current models generally present acceptable discriminative capacity, with C/AUC statistics between 0.75 and 0.82. Interestingly, although machine learning-based models are increasingly frequent, their predictive advantage over traditional statistical models is modest. This meta-analysis found a pooled C-statistic of 0.75 (95% CI: 0.73–0.77) for traditional regression models vs. 0.76 (95% CI: 0.72–0.79) for machine learning models, with extremely high heterogeneity (I^2^ = 99.9%), suggesting that technical sophistication does not necessarily translate into better predictive capacity.

From a historical perspective, HTN prediction has traversed different methodological eras. The first generation of models, exemplified by the Framingham HTN Risk Score (24), established the paradigm of logistic regression-based equations, with a C-statistic of 0.788. This model, which remains the most externally validated, established the standard against which subsequent approaches are compared, demonstrating its robustness in various populations, from the Whitehall II study in the United Kingdom (C-statistic 0.80) (25) to validations in multiethnic American populations (MESA, C-statistic 0.77). Between 2010 and 2015, region-specific scores proliferated for various populations, maintaining a traditional logistic regression approach, while from 2015 onwards, the first applications of machine learning algorithms such as Random Forest and SVM emerged, although initially with modest benefits in terms of predictive capacity (26, 27).

The current era (2020-present) is dominated by models using deep neural networks and advanced algorithms such as XGBoost. Recent studies such as Ji et al. (19) have developed models with XGBoost reaching an AUC of 0.893 in a massive cohort of more than 4.2 million participants, while Kanegae et al. (28) reported an AUC of 0.877, superior to the 0.859 of logistic regression in the same population. This increase in performance comes with greater interpretative complexity, which studies such as Elshawi et al. (29) have attempted to mitigate through explainability techniques such as SHAP, which allow the identification of key risk factors and their interactions.

The added value of new methodological approaches does not primarily lie in better pure discriminative capacity, but in their ability to handle complex non-linear relationships and efficiently process large volumes of heterogeneous data. Algorithms such as Random Forest and XGBoost capture complex interactions between variables without specifying them beforehand, especially useful in multifactorial conditions such as HTN. Additionally, incorporating explanatory techniques such as SHAP and LIME provides details on individual risk factors, enhancing personalized medicine and facilitating clinical interpretation. However, they still present greater technical complexity than traditional models (17, 22, 30, 31).

A critical limitation identified in our review is the marked geographical concentration of included studies, with all models developed exclusively in Asian (China, Korea, Bangladesh) and Middle Eastern populations (Qatar, United Arab Emirates). This SR identified no cross-sectional HTN diagnostic models from Latin America, sub-Saharan Africa, or Eastern Europe—regions that collectively bear a substantial HTN burden and where population-based screening tools would be most valuable.

The absence of Latin American models is particularly concerning given the regional HTN prevalence of 42.5% in the Southern Cone, as documented by Carrillo-Larco et al. (32) in their systematic analysis of cardiovascular models in the region. Their research revealed that despite high disease burden, no cross-sectional predictive models have been specifically developed or validated for Latin American populations. Similarly, sub-Saharan Africa presents a paradoxical scenario: HTN prevalence ranges from 30%–45% in many countries, access to blood pressure monitoring is severely limited in rural and peri-urban settings, yet no diagnostic models have been developed for these populations. Eastern European countries, which have experienced epidemiological transitions distinct from those in Western Europe, also lack region-specific models, despite documented differences in cardiovascular risk profiles.

This geographical concentration raises fundamental questions about the external validity and applicability of existing models. Ethnic, dietary, genetic, and socio-environmental differences characterize underrepresented populations and could significantly modify the relative weight of traditional predictors. Rubinstein et al. (33) found that the distribution patterns of cardiovascular risk factors in Latin America differ considerably from those in Asian or Caucasian populations, potentially limiting the validity of models developed elsewhere when applied to Latin American contexts. Miranda et al. (34) demonstrated that social and economic factors play a particularly significant role in HTN epidemiology in Latin America, variables that are frequently underestimated in models from other regions. Similar considerations apply to African populations, where genetic polymorphisms associated with salt sensitivity, dietary patterns rich in carbohydrates and low in potassium, and high prevalence of obesity create a distinct risk profile.

Whether the absence of models in our review reflects true research gaps or limitations in published literature accessibility (language barriers, journal indexing, grey literature), the practical consequence remains: clinicians and public health practitioners in these regions lack validated, contextually appropriate diagnostic tools. This represents not only a limitation in current knowledge but an urgent call for future research. Developing and validating region-specific cross-sectional HTN models for Latin America, sub-Saharan Africa, and Eastern Europe should be research priorities, particularly given the feasibility of leveraging existing large-scale health surveys (such as ENDES in Peru, NHANES equivalents in other countries) to develop these tools at relatively low cost.

### 4.3 Critical analysis of predictor variables

In the analysis of cross-sectional models for HTN diagnosis, the consistent presence of five fundamental variables that appear in more than 70% of the models stands out. Due to its direct relationship with arterial stiffening, age is the most ubiquitous predictor in more than 90% of the analyzed models. Aging causes progressive structural changes in vascular walls, including increased collagen deposition, reduced elastin content, endothelial dysfunction, and alterations in calcium homeostasis within vascular smooth muscle cells. These age-related vascular changes directly increase peripheral resistance and reduce arterial distensibility, contributing to elevated systolic blood pressure and explaining why isolated systolic HTN is predominant in older adults (35, 36).

BMI constitutes the second most important predictor (85%–90% of models) and contributes to HTN through multiple interrelated pathways, including sympathetic nervous system activation, stimulation of the renin-angiotensin-aldosterone system, promotion of insulin resistance, proinflammatory states, and potential renal compression. Research demonstrates that each unit increase in BMI is associated with approximately 0.8–1.7 mmHg of increase in systolic blood pressure, with stronger associations in men (36, 37). Body fat distribution patterns, particularly central adiposity, show a stronger correlation with HTN risk than BMI alone, which explains the inclusion of measures such as waist circumference in models like AlKaabi's (17) for the Qatari population, where this measure achieved greater diagnostic accuracy than overall BMI.

Sex (75%–80% of models) is a significant predictor due to hormonal differences and variations in body composition between men and women. The BMI-blood pressure relationship is approximately twice as strong in men as in women. HTN patterns differ notably throughout life, with men showing higher prevalence at younger ages and women after menopause (38, 39). Specifically, studies such as Everett and Zajacova's demonstrated that these differences remain even after adjusting for multiple risk factors, suggesting underlying gender-specific biological mechanisms, including hormonal effects on vascular function and differences in sympathetic nervous system activity (39). Including gender as a predictor variable is particularly relevant for population screening tools, as it enables more precise risk stratification.

The presence of diabetes (70%–75% of models) contributes to HTN through insulin resistance, formation of advanced glycation end products, endothelial dysfunction, oxidative stress, deregulation of the renin-angiotensin-aldosterone system, and sodium retention, with a 1.5–2.5 times higher risk of presenting HTN compared to non-diabetic individuals (40). Ji et al.'s study showed that diabetes alone had a predictive weight comparable to age, justifying its inclusion in simplified diagnostic models (19). Notably, several models incorporate not only the dichotomous presence of diabetes but also indicators of glycemic control, such as HbA1c levels. However, simplified versions usually maintain only the categorical variable for applicability reasons.

Despite substantial evidence linking social determinants with HTN risk, most traditional cross-sectional models have excluded these variables, potentially undermining their diagnostic accuracy, particularly for disadvantaged populations. Relevant social determinants include educational level, income, employment status, health insurance coverage, neighborhood characteristics, housing quality, food environment, social isolation/support, and racial/ethnic origin. These factors influence HTN through behavioral pathways (affecting diet, physical activity, and medication adherence), psychosocial stress activation, barriers to healthcare access, and cumulative disadvantages throughout life (41, 42). Mahmoud et al.'s study (18) in the United Arab Emirates was notable for incorporating socioeconomic characteristics, achieving a significant improvement in diagnostic validity among specific population subgroups (43).

The incremental value of laboratory biomarkers represents a third dimension in HTN diagnostic models. Still, their added value beyond clinical variables deserves critical examination, particularly considering cost-effectiveness and accessibility. Commonly used biomarkers include urinary albumin-creatinine ratio (UACR), C-reactive protein (CRP), serum aldosterone, B-type natriuretic peptide (BNP/NT-proBNP), growth differentiation factor-15 (GDF-15), microRNAs, and chitinase-3-like protein 1 (CHI3L1). These markers reflect underlying pathophysiological processes, including early renal dysfunction, inflammation, neurohumoral activation, and vascular stress (44, 45).

Additional laboratory biomarkers typically improve discriminative capacity by modest margins, with AUC improvements ranging from 0.02 to 0.04 beyond clinical variables alone. Combinations of complementary biomarkers provide greater improvement than individual markers, although still relatively limited in absolute terms. The cost-effectiveness of incorporating biomarkers varies significantly depending on the target population, healthcare system context, and time horizon. UACR represents a practical, relatively inexpensive biomarker with proven incremental value, while cardiac biomarkers such as BNP and GDF-15 may be more valuable in patients with multiple cardiovascular risk factors (46, 47).

The most definitive finding on the value of biomarkers comes from studies that directly compare models with and without these markers. Ji et al.'s comprehensive study (19) with 4,287,407 participants compared “laboratory-free” vs. “semi-laboratory” HTN diagnostic models using extreme gradient boosting decision tree algorithms. The results were surprising: the AUC for the laboratory-free model was 0.893 compared to 0.894 for the model with laboratory tests. The authors concluded that “blood analysis factors had little effect on improving the HTN prediction model” (19). Chowdhury et al.'s SR and meta-analysis examining 52 articles on HTN prediction models found minimal differences in performance between models with and without biomarkers, with a pooled C-statistic of 0.75 for traditional regression models and 0.76 for models primarily using biomarkers, a statistically significant but clinically questionable improvement (48).

From a cost-effectiveness perspective, the marginal performance gains provided by laboratory biomarkers are unlikely to justify the associated costs and logistical barriers, particularly in resource-limited settings. Laboratory testing requires infrastructure (including equipment, reagents, and quality control systems), trained personnel, reliable supply chains, and sample processing capacity—substantially increasing per-person screening costs compared to clinical-only models. For population-level screening programs where the goal is identifying individuals who warrant confirmatory blood pressure measurement, investing resources in biomarkers that provide <0.02 AUC improvement represents inefficient resource allocation. Moreover, requiring laboratory access creates barriers that preferentially exclude those at highest risk due to limited healthcare access, fundamentally undermining the equity objectives of screening programs. The principle of proportionate universalism suggests that screening strategies should be accessible across all socioeconomic strata; mandatory laboratory testing, however, contradicts this principle by creating a two-tier system where screening availability depends on the availability of laboratory infrastructure.

The critical understanding of predictor variables in cross-sectional HTN models reveals that fundamental clinical predictors (age, BMI, sex/gender, and diabetes status) constitute the most robust pillars for effective diagnosis, thanks to their solid pathophysiological basis and universal accessibility. Regarding social determinants, their incorporation presents a dilemma: although their inclusion could improve diagnostic accuracy in certain contexts, it must be implemented cautiously to avoid inadvertent stigmatization or discrimination of specific populations. The true utility of these factors lies in guiding equitable health policies rather than labeling individuals according to their socioeconomic or geographical condition. As for laboratory biomarkers, their modest improvement in predictive performance hardly justifies their inclusion in population screening tools, as it contradicts the fundamental purpose of these models: to reach people with limited access to health services. As you correctly point out, if an individual already has access to a laboratory, performing a blood pressure measurement would be more efficient and direct. Therefore, the most valuable models will be those based exclusively on self-reported variables or easily measurable ones in community settings, prioritizing simplicity and accessibility over marginal increases in diagnostic accuracy.

### 4.4 Implications for clinical practice

The practical implementation of cross-sectional HTN diagnostic models requires prioritizing tools based on self-reportable or easily measurable variables (age, sex, BMI, family history, diabetes) that avoid dependence on biochemical markers. Simplified formats such as visual nomograms and brief questionnaires are more likely to be adopted by healthcare professionals and end users compared to complex algorithms. The ideal model must balance diagnostic accuracy with real implementability, recognizing that a theoretically optimal but complex instrument has less population impact than a moderately accurate but widely usable one. “Laboratory-free” models with AUCs close to 0.80 can be applied in community settings without requiring specialized equipment, making them particularly suitable for resource-limited contexts. Integration with preexisting electronic record systems is crucial for sustainable adoption, enabling seamless risk assessment during routine encounters without significantly increasing the time required for clinical workflows.

Cross-sectional models are most valuable when implemented as the first stage of a two-step screening approach, where models identify high-risk individuals who then undergo confirmatory blood pressure measurement. This strategy could potentially reduce operational costs compared to universal screening, particularly in resource-limited settings where blood pressure monitoring equipment and trained personnel are scarce. However, the efficiency of targeted screening depends on model calibration and cost-effectiveness, which vary across healthcare contexts. Economic evaluations comparing different screening strategies in specific country contexts are needed but currently lacking. Such analyses should incorporate costs of model implementation, confirmatory testing, and subsequent treatment against benefits of earlier detection and cardiovascular event prevention.

Despite their potential utility, implementing these models faces considerable barriers in LMICs, where they would paradoxically be most beneficial. Key limitations include lack of validation in diverse populations, as most models have been developed with data from Asian or Caucasian populations, with limited representation of Latin American or African populations. Ethnic heterogeneity within and across countries may require multiple models or local adaptations to maintain diagnostic accuracy. Additionally, workforce limitations in rural primary care centers, including insufficient personnel trained in risk assessment tools, and instability of technological infrastructures represent significant obstacles. Data quality in routine primary care settings may not match research study standards, potentially degrading model performance. Although basic infrastructure requirements are minimal, they still present challenges. Standardized protocols for height and weight measurement are crucial, as BMI calculation errors can significantly impact model performance. Healthcare systems must also establish clear referral pathways for individuals classified as high-risk, ensuring that screening leads to confirmatory measurement and appropriate management. Effective implementation requires that both healthcare providers and community members understand the purpose and limitations of these screening tools. Educational materials adapted to different levels of health literacy are essential, allowing patients to understand and interact with their risk profile, thus fostering greater adherence to preventive recommendations.

Incorporating these models into clinical guidelines and primary care protocols has been inconsistent. Most international guidelines still do not include specific recommendations on using cross-sectional models for HTN diagnosis. This gap is mainly due to methodological heterogeneity among studies and limited evidence on long-term cost-effectiveness and real-world implementation outcomes. To maximize impact, it is essential to establish standardized action thresholds and clear care pathways. Defining risk levels (low, intermediate, and high) with specific recommendations for each category could improve clinical decision-making and reduce interprofessional variability. Equally important is continuous post-implementation evaluation to assess model performance in real-world settings and allow for local calibration when needed. Healthcare worker buy-in is critical—models perceived as adding work without clear benefit are unlikely to be consistently applied. Important enablers include integration with existing electronic health records, endorsement by national health authorities or professional societies, and alignment with ongoing non-communicable disease screening programs.

The gap between model development and real-world implementation represents a critical challenge. Successful adoption requires not only technical validation but also careful consideration of organizational, training, and cultural aspects specific to each healthcare context. Future research should prioritize implementation science studies that evaluate not only whether models work in practice but also how to optimize their integration into diverse healthcare contexts. Hybrid effectiveness-implementation trials could assess both clinical outcomes (cases detected, blood pressure control achieved) and implementation outcomes (adoption, fidelity, sustainability, cost). Particular attention should be given to LMICs, where the need is greatest but implementation research is scarcest. Additionally, participatory approaches involving end-users (healthcare workers, patients, policymakers) in model selection and adaptation could improve contextual fit and likelihood of sustained implementation.

### 4.5 Strengths and limitations

Among the main strengths of this SR is its rigorous methodological approach, which included an exhaustive search in multiple databases, a standardized and blind peer selection process, and a systematic assessment of risk of bias using the PROBAST tool specific for predictive models. The exclusive inclusion of cross-sectional models for HTN diagnosis filled a gap in the literature, differentiating itself from previous reviews focused on prognostic models. Likewise, the detailed analysis of predictor variables and their frequency of inclusion provides valuable information for future developments. At the same time, the comparative assessment of the incremental value of biomarkers vs. simplified models offers practical guidance for resource-limited contexts. The geographical diversity of the included models, albeit with limitations, allows for identifying global patterns in developing these diagnostic tools and their potential applicability in different healthcare contexts.

The limitations of this review include significant heterogeneity among studies in terms of design, included variables, operational definitions of HTN (≥140/90 mmHg vs. ≥130/80 mmHg), and reported performance metrics, which prevented conducting a formal meta-analysis. While all studies consistently reported AUC or C-statistic, other important performance metrics such as sensitivity, specificity, positive and negative predictive values, and calibration statistics were variably reported or absent across studies, limiting comprehensive cross-model comparisons beyond discriminative capacity. The unbalanced geographical representation, with a predominance of Asian studies and a scarcity or complete absence of research from Africa, Latin America, and certain parts of Europe, limits the generalizability of findings to these populations. Variable methodological quality was also observed, with frequent deficiencies in the reporting of calibration and reclassification, as well as scarce independent external validation, particularly in older studies. Most models lack impact assessments that demonstrate tangible clinical benefits after implementation, and there is limited information on cost-effectiveness and acceptability among health professionals and patients. Finally, although relevant social factors for HTN diagnosis were identified, their incorporation into predictive models remains insufficient, perpetuating potential biases against vulnerable populations.

## 5 Conclusions and recommendations

Cross-sectional predictive models for HTN diagnosis represent valuable tools to optimize case detection in various healthcare contexts, demonstrating generally acceptable discriminative capacity with AUC/C statistics between 0.75 and 0.89. The critical analysis of predictor variables reveals a consistent core of predominant factors (age, BMI, sex/gender, and diabetes) that constitute the axis of most models, regardless of their methodological complexity. Incorporating laboratory biomarkers provides marginal benefits (improvements of 0.02–0.04 in AUC) that hardly justify the incremental costs and implementation barriers, especially in resource-limited contexts. This review evidences critical geographical gaps, with a concerning absence of validated models for Latin American and African populations despite the high prevalence of undiagnosed HTN in these regions. This gap represents an urgent public health priority, as millions of individuals in under-resourced settings remain undiagnosed and without access to early intervention.

We recommend prioritizing the development and external validation of specific models for underrepresented populations, particularly in Latin America, where prevalence is high and risk factors present distinctive patterns. Future models should incorporate social determinants relevant to the local context, variables frequently omitted despite their demonstrated ability to improve diagnostic accuracy and reduce biases against vulnerable populations. To optimize implementation and maximize population-level impact, we recommend developing simplified tools without biomarkers that maintain adequate diagnostic capacity while maximizing applicability in community and primary care settings where diagnostic resources are scarce. It is essential to establish rigorous methodological standards, with adherence to TRIPOD guidelines and comprehensive evaluation that includes discrimination, calibration, reclassification, and clinical impact. Finally, we suggest developing accessible presentation formats (nomograms, digital calculators, mobile applications) that facilitate adoption by healthcare professionals and patients, accompanied by strategies that accelerate the identification of undiagnosed hypertensive individuals who urgently require intervention.

## Data Availability

The original contributions presented in the study are included in the article/Supplementary Material, further inquiries can be directed to the corresponding author.
